# The effect of tillage systems on phosphorus distribution and forms in rhizosphere and non-rhizosphere soil under maize (*Zea mays L*.) in Northeast China

**DOI:** 10.1038/s41598-020-63567-7

**Published:** 2020-04-20

**Authors:** Thidaphone Xomphoutheb, Shuai Jiao, Xinxin Guo, Frank Stephano Mabagala, Biao Sui, Hongbin Wang, Lanpo Zhao, Xingmin Zhao

**Affiliations:** 1 College of Resources and Environment, Jilin Agricultural University, Changchun, 130118 P.R. China; 2 Department of Environmental Engineering, Faculty of Engineering and Green Technology, Universiti Tunku Abdul Rahman, Kampar, 31900 Malaysia

**Keywords:** Ecology, Ecology, Environmental sciences

## Abstract

An appropriate tillage method must be implemented by maize growers to improve phosphorus dynamics in the soil in order to increase phosphorus uptake by plant. The objective of this study was to investigate the effects of tillage systems on phosphorus and its fractions in rhizosphere and non-rhizosphere soils under maize. An experimental field was established, with phosphate fertilizers applied to four treatment plots: continuous rotary tillage (CR), continuous no-tillage (CN), plowing-rotary tillage (PR), and plowing-no tillage (PN). Under the different tillage methods, the available P was increased in the non-rhizosphere region. However, the concentration of available P was reduced in the rhizosphere soil region. The soil available P decreased with the age of the crop until the maize reached physiological maturity. The non-rhizosphere region had 132.9%, 82.5%, 259.8%, and 148.4% more available P than the rhizosphere region under the CR, PR, CN, and PN treatments, respectively. The continuous no-tillage method (CN) improved the uptake of soil phosphate by maize. The concentrations of Ca_2_-P, Ca_8_-P, Fe-P, Al-P and O-P at the maturity stage were significantly lower than other seedling stages. However, there was no significant relationship between total P and the P fractions. Therefore, a continuous no-tillage method (CN) can be used by farmers to improve phosphorus availability for spring maize. Soil management practices minimizing soil disturbance can be used to impove phosphorus availability for maize roots, increase alkaline phosphatase activity in the rhizosphere soil and increase the abundance of different phosphorus fractions.

## Introduction

Maize (*Zea mays*), also known as corn, is an important cereal crop grown under varied climatic conditions. China is the second-largest producer of maize after the United States. Maize can be processed into human food, animal feed, and industrial products. It is a high-production crop of national importance. A soil environment conducive to growth is required for the optimum growth and yield of maize. Tillage methods in cropping systems have been a part of most agricultural systems throughout history^1^. The tillage method has a significant effect on the yield components of maize^2^. Tillage changes soil physical properties, such as its water-holding capacity, pore size distribution, bulk density, and aggregation. Tillage allows more organic matter to be degraded by microorganisms, while no-tillage systems promote the establishment and strengthening of macroaggregates^3–5^. An appropriate tillage system is necessary to provide an appropriate environment for seed germination, weed control, regular moisture availability and the reduction of surface runoff through increased infiltration^6–8^. Adel El Titi^1^ explained the importance of understanding the structures and functions of soil ecosystems under different tillage practices as a crucial requirement for applying farming concepts. However, intensive maize production has recently resulted in some adverse effects to the soil, such as nitrate leaching^2^.

Phosphorus is an essential element for maize growth. The two vital processes in the transfiguration and translocation of phosphorus elements in the soil are geochemical and biological^9^. Soil phosphorus (P) exists dynamically as dissolvable, labile, and non-labile P, and the chemical equilibrium between labile and non-labile P is weaker than the balance between dissolvable and labile P^10^. Phosphorus fertilizer use has played an essential role in increasing crop productivity^11^. Phosphorus, mainly organic phosphorus, is only available for plant uptake after hydrolysis by the enzyme phosphatase^12^. Adequate phosphorus fertilization can affect maize plant height, leaf area index, kernel number, leaf photosynthesis, and plant growth^13,14^. However, the excessive phosphate fertilizer used in agricultural production becomes a source of surface water pollution. Therefore, to maintain sustainable agricultural development and protect the environment, it is vital to establish proper tillage and fertilization methods in order to reduce fertilizer loss in agricultural production, the availability of phosphorus is dependent on the production system applied^15^. Tillage methods can change the phosphorus retention parameters in the near-surface zone^15^. The spatial distribution of phosphorus can also affect its availability, especially in rhizosphere and non-rhizosphere soil. Rhizosphere soil refers to the narrow soil, which is directly influenced by root secretions and their affiliated soil microorganisms^16^. The zone of soil around the roots where the soil properties, soil microorganisms, and plant roots interact is abundant in organic compounts^17–20^. Dynamic changes in plant nutrients, biology, and soil chemistry take place in the rhizosphere. The enzyme activity in rhizosphere soils is generally higher than that in non-rhizosphere soil^21^. Various techniques for studying the chemical changes in rhizosphere soil have been established for annual crops, grasses, and legumes^22^. Guo^23^ demonstrated that the continuous cropping of maize and soybean in different tropical soils treated with a high dose of P fertilizer greatly reduced the labile and moderately labile inorganic (Pi) fractions. P in the soil has unique properties, such as low solubility and high fixation by soil particles. Therefore, the available P for crops is controlled by two factors: (1) the bioavailability and acquisition of P based on rhizosphere processes and (2) the limited availability and acquisition of P in terms of plant root architecture as well as mycorrhizal association^24^. To estimate the various P fractions and P changes in soil, different fractionation methods with multiple chemical sequential extractions can be used^25,26^. (Safari Sinegani)^27^ reported that plants significantly decreased the levels of all inorganic P fractions in the rhizosphere soil compared to those in non-rhizosphere soil. The reduction was not equal for each fraction, and the percentage of apatite-P increased in the rhizosphere soil. (Yong-Fu)^28^ also noted a significant decrease in P fractions, pH and phosphatase activities in the rhizosphere. Soluble P in solution in the rhizosphere should be exchanged 20 to 50 times per day by P delivery from bulk soil to the rhizosphere to meet crop requirements^29^. Numerous studies have focused on the changes in the phosphorus fractions associated with soil chemical properties under different types of fertilizers^30^.

Previously, many studies have been conducted on how different tillage systems can be used as tools for increasing the crop yields. There is little concentration about which tillage method is suitable for maintaining a supply of available P for maize, thereby decreasing the fertilizer application and improving the environment. We hypothesized that tillage systems have an effect on phosphorus and its forms in the rhizosphere and non-rhizosphere soil. Therefore, the objective of this study was to assess the effects of different tillage systems on the distribution of phosphorus and its different forms in the rhizosphere and non-rhizosphere soil under maize (*Zea mays L*.) in northeastern China.

## Results

### The available P content in the maize rhizosphere and non-rhizosphere soil under different tillage systems

**Figure 1:**
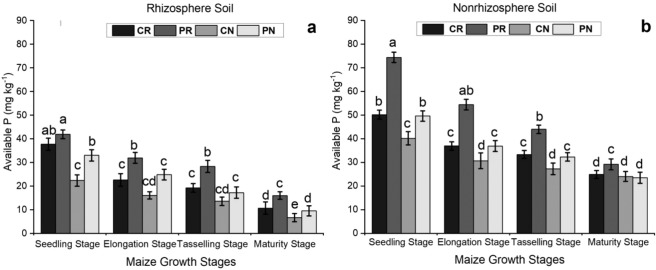
Comparison of available P in the maize rhizosphere and non-rhizosphere under different tillage methods. The bars represent the standard error of the three replicates. The letters above the columns represent significant differences among the four tillage methods.

The available soil P was evaluated under the different tillage methods in the rhizosphere and non-rhizosphere soil. The available P significantly decreased with the age of the maize., which indicated that much more nutrients were taken by plant roots with maize growing. Then, the available P continually declined with crop growth. The rhizosphere region showed a lower content of available P than the non-rhizosphere region (Fig. 1). Compared to the rhizosphere soil, the non-rhizosphere soil had 132.9%, 82.5%, 259.8%, and 148.4% more available P in the CR, PR, CN and PN treatments, respectively, at the maturity stage. The CN treatment had the lowest amount of available P and the PR treatment had the highest amount of available P throughout the growth stage, which indicated that soil disturbance was beneficial to the increase of available phosphorus content. (Fig. 1).

### The total content of P in the maize rhizosphere and non-rhizosphere soil under different tillage systems

**Figure 2:**
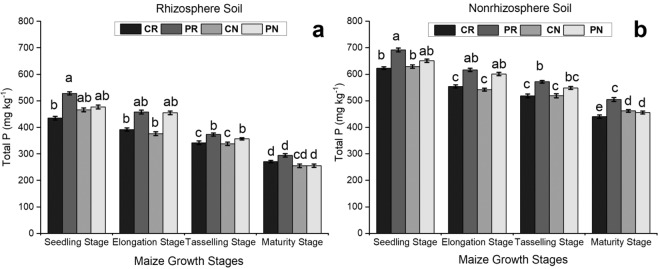
Comparison of total P in the maize rhizosphere and nonrhizosphere soil under different tillage methods. The bars represent the standard error of the three replicates. The letters above the columns represent significant differences among the four tillage methods.

The total P concentration in the soil decreased with the maize growing up in both soils (Fig. 2). At the early stage of plant growth, the level of total P in both soils was high. Afterward, it decreased with time until harvesting. In comparison to the rhizosphere soil, the non-rhizosphere soil had 62%, 71.4%, 80.9% and 78.3% more total P in the CR, PR, CN and PN treatments, respectively. The CN treatment had the lowest amount of total P throughout the growth stages (Fig. 2).

### Alkaline phosphatase content in the maize rhizosphere and non-rhizosphere soil under different tillage systems

There were significant differences in the alkaline phosphatase level between the maize growth stages. At the seedling stage, all treatments contained a higher level of alkaline phosphatase. This level decreased with the age of the maize until the maturity period, which marked the lowest amount of alkaline phosphatase in both soils studied. Compared to the rhizosphere soil, the non-rhizosphere soil had 106.4%, 57.8%, 71.8% and 54% more alkaline phosphatase in the CR, PR, CN and PN treatments, respectively. The CN treatment showed the lowest amount of alkaline phosphatase over the whole growing cycle. All these results showed that there was an increase in chemical phosphatase activity in both regions of the studied soil, and the CN treatment showed lower alkaline phosphatase levels compared to the other treatments.

### The distribution of P fractions in the maize rhizosphere and non-rhizosphere soil under different tillage systems

Figure 4(a–e) shows the different levels of P fractions in the maize rhizosphere and non-rhizosphere soil at the seedling and maturity stages of maize growth. The amounts of Ca_2_-P (Fig. 4a), Ca_8_-P (Fig. 4b), Fe-P (Fig. 4d) and O-P (Fig. 4e) were high at the seedling stage and decreased at the maturity stage. There were significant differences in the amounts of Ca_2_-P, Ca_8_-P, Fe-P, and O-P under the different tillage methods at different growth stages. The seedling stage had the maximum amounts of Ca_2_-P, Ca_8_-P, Fe-P and O-P fractions compared with those amounts in the other treatments (Fig. 4a). The rhizosphere soil displayed lower amounts of Ca_2_-P, Ca_8_-P, Fe-P and O-P than the non-rhizosphere soil in the two soil regions studied (Fig. 4b).

**Figure 3:**
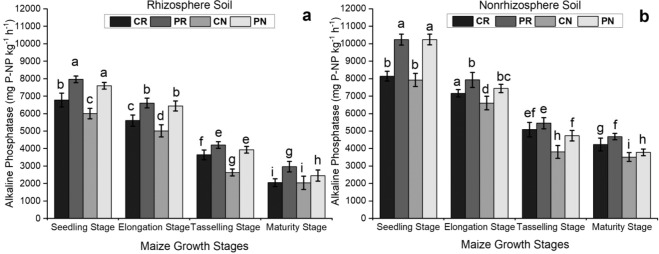
Comparison of alkaline phosphatase levels in the maize rhizosphere and nonrhizosphere soil under different tillage methods. The bars represent the standard error of the three replicates. The letters above the columns represent significant differences among the four tillage methods.

**Figure 4:**
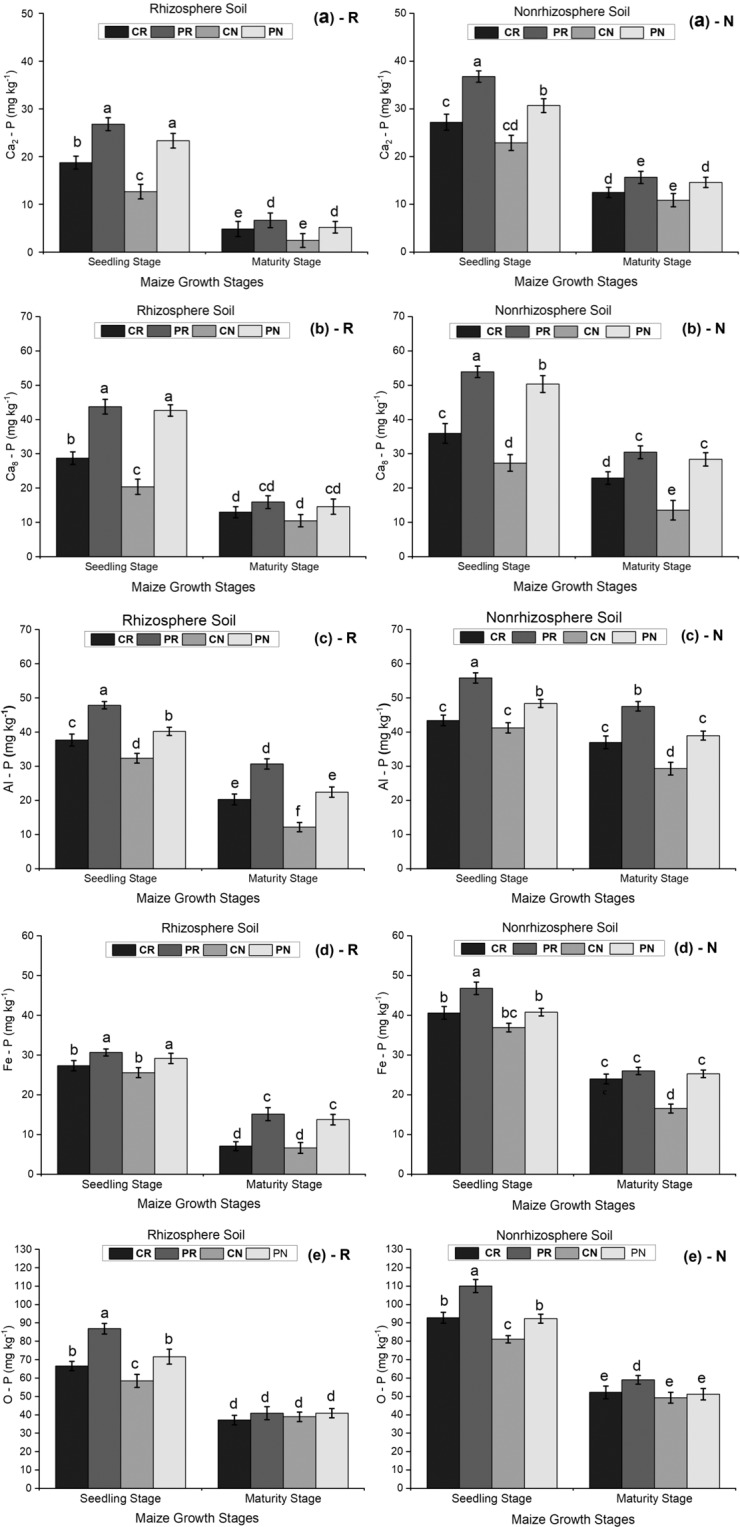
(**a–e**) The concentrations of P fractions in the maize rhizosphere and non-rhizosphere soil at the seedling and maturity stages of maize growth.

The Pearson correlation coefficient (Table 1) showed that the available P in the soil was significantly positively correlated with Ca_2_-P(r = 0.871), Ca_8_-P (r = 0.910), Al-P(r = 0.696), Fe-P(r = 0.759), and occluded-P(r = 0.844). There was no significant relationship between the total P and the P fractions found. The total P measured in the soil had no relationship with the evaluated P fractions.

**Table 1:** The correlation coefficients (r) of the relationships between selected Pfractions and total phosphorus (mg kg^−1^).

	Total P	Available P	Ca_2_-P	Ca_8_-P	Al-P	Fe-P	O-P
Total P	1.000						
Available P	0.179	1.000					
Ca_2_-P	−0.103	0.871**	1.000				
Ca_8_-P	0.042	0.910**	0.927**	1.000			
Al-P	0.526	0.696**	0.549*	0.672**	1.000		
Fe-P	0.002	0.759**	0.945**	0.875**	0.593*	1.000	
O-P	−0.048	0.844**	0.976**	0.877**	0.498	0.940**	1.000

* and ** indicate significance at p < 0.05 and p < 0.01, respectively.

## Discussion

### Dynamics of soil available P and total P during maize growth

The rhizosphere soil is dominated by microbes and enzymes^31^. The rhizosphere is modified by plant roots, and has more microbial communities than bulk soil or non-rhizosphere soil^32^. The application of any compound used as fertilizer to a plant of interest, such as maize, can influence the microbial activity and community structure in the rhizosphere soil^16^. There were significant differences in the amounts of available and total P during the growth stages of maize (Figs. 1 and 2). For the different treatments, it appeared that the contents of total and available P were higher during the seedling stage in both the rhizosphere and the non-rhizosphere soil. Both P types continued to decrease with plant growth until the harvesting period. This decrease in nutrients was probably due to uptake by the growing crop.The nutrient concentration in non-rhizosphere soil was significantly higher than that in rhizosphere soil during the full summer maize growing season^33^, which was consistent with our results. However, it was similar to our findings that the addition of P and K fertilizers significantly increased the levels of microelements (P, K, Ca, and Mg) in rhizosphere soil. Additionally, the fertilizers transformed the pH and electrical conductivity of the rhizosphere soil compared to those in the control^34^. Rhizosphere and non-rhizosphere soil are essential soil regions that connect the soil environment to plant root systems. A lack of soil disturbance during soil preparation can influence the availability of phosphorus nutrients for adsorption by maize. The application of phosphorus significantly altered the amount of available P in non-rhizosphere soil under all maize tillage methods applied in this research.

### The available P and total P content in the maize rhizosphere and non-rhizosphere soils under different tillage systems

Phosphorus (P) is an essential mineral nutrient for plant growth and development, and it is affected by different systems of physical soil manipulation^35,36^. The mechanical management of soil during tillage may increase the contact chances between soil solution- or fertilizer-derived P and soil particles. It exposes soil particles, enabling the establishment of fixed, insoluble P compounds^37^. Tillage methods may influence the availability and distribution of plant nutrients (N, P, K)^38–41^. No-tillage systems affect some chemical parameters associated with soil acidity that may influence P accessibility, plant growth, and yield^42^. In our investigation, rhizosphere soil had lower available and total P than non-rhizosphere soil.The low amount of P in the rhizosphere was due to the rapid uptake of P by plant roots, which was facilitated by the low solubility and mobility of P in the soil^24^. The continuous no-tillage treatment (CN) had the lowest concentration of phosphorus in the maize rhizosphere soil, while plowing-rotary tillage (PR) had the highest level of phosphorus in both soil regions. This indicated that less soil disturbance promoted the availability and uptake of phosphorus by the plants; the no-till method maintains a favorable soil environment, and therefore, phosphate nutrients are readily available for uptake by the roots (Figs. 1 and 2). The continuous no-tillage practice significantly improved the soil’s ability to hold phosphates in the non-rhizosphere soil and thus resulted in an increasing amount of soil P under the CN treatment compared to that under the rotary tillage systems. Similar results were also reported by Zhang^43^, who suggested that short-term no-tillage improved P availability in surface soils at 0–20 cm. The addition of phosphorus through manure application also increased levels of different phosphorus forms and the phosphorus saturation of the near-surface soil zone in a no-tillage system^44^. These findings indicate that the no-tillage method in spring maize has significant effects on phosphorus availability and improve our understanding of P management practices.

### Alkaline phosphatase activity in the soil under different tillage systems

Alkaline phosphatase is secreted by bacteria, fungi, and earthworms, and it works catalytically at a high pH of 7.00. The abundance of different mineralized phosphate groups can be predicted using alkaline phosphatase. Alkaline phosphatase speeds up or slows down the cleavage of ester-phosphate bonds, making P available in soils^45,46^. Acid and alkaline phosphatases in the rhizosphere and bulk soils of legumes have various functions, including Nfixation in beans^47^. In the rhizosphere and non-rhizosphere soils, alkaline phosphatase was more active in the continuous no-tillage treatment than in the other three treatments. Continuous plowing-rotary tillage was observed to have the lowest alkaline phosphatase activity. The tillage method had a significant effect on the alkaline phosphatase balance in the soil (Fig. 3). The level of alkaline phosphatase decreased with the age of the maize, and the lowest amount of alkaline phosphatase in both soils was observed at the maturity stage. In comparison to the non-rhizosphere soil, the rhizosphere soil showed slightly lower alkaline phosphatase concentrations. The CN treatment showed the lowest alkaline phosphatase level of all treatments over the whole growing cycle. Compared with the CN treatment, there was an increase in alkaline phosphatase activity in the other three treatments. This suggested that no-tillage may influence the availability of water and improve the amount and variety of organisms in the soil. Therefore, no-tillage increased the activity of alkaline phosphatase in the soil. It was also found that alkaline phosphatase was more active at the tasseling stage than at the other maize growth stages evaluated in all the treatments (Fig. 3). Alkaline phosphatase works in the presence of phosphate nutrients. During the reproductive growth stage, it is necessary to increase the availability of phosphate in the soil. NTSM (no-tillage with corn straw return) and NTG (no-tillage with grass) increased P content and phosphatase enzyme activity and provided a basis for using this method to improve P availability and decrease the application of fertilizer to soils^36^. A similar observation also indicated that alkaline phosphatase activity in rhizosphere soil was significantly higher than that in non-rhizosphere bulk soil^47^. The use of phosphate fertilizer may increase alkaline phosphatase activity in the rhizosphere soil. Overall, the continuous no-tillage method facilitated more alkaline phosphatase activities in the maize rhizosphere soil. The soil under plowing with rotary tillage had the lowest soil enzyme components compared to that under the other tillage methods; therefore, the CN treatment could be used as a strategy for soil health and productivity, resulting in a sustainable agricultural system.

### The partitioning of P fractions in the maize rhizosphere and non-rhizosphere under different tillage systems

The Hedley sequential-phosphorus (P) fractionation method has been used worldwide to investigate the effects of land-use and management systems on soil P. In natural environments, vegetation varieties, composition, and percent of vegetation cover significantly affect all P fractions. Most P fractions increase with the level of phosphorus applied^25^. There were significant correlations between total P and Ca_2_-P, Ca_8_-P, and Al-P, and the relative abundances of P forms were in the order of Ca_10_-P > Ca_8_-P > Al-P > Fe-P > Ca_2_-P > Occl-P^48^.Compared to those under conventional tillage, the amounts of organic matter and phosphorus in the top few centimeters under no-tillage were higher^49^. The evaluation of the relationship between P availability indicators and inorganic P fractions showed the abundances of the different P forms, which were in the order of Ca_2_-P < Fe-P < Al-P < Occluded-P < Ca_8_-P < Ca_10_-P. Total P was positively correlated with Olsen P and exchangeable P^50^. In both soils, the amounts of the different P fractions were high at the seedling stage and then decreased gradually until maturity. P fractions are associated with phosphate nutrients, which are required by the plant during its whole life cycle; this is why the P fractions were abundant at the seedling stage (Fig. 4a–e). The levels of the P forms in the maize rhizosphere and non-rhizosphere soil followed this arrangement: CN < CR < PN < PR treatments. However, the non-rhizosphere soil had higher levels of the different P fractions than the rhizosphere soil. The no-tillage method caused less soil disturbance, resulting in a healthy soil environment with good soil aeration and soil moisture levels. However, our analysis showed that available P was positively correlated with the Ca_2_-P, Fe-P and Al-P, Ca_8_-P, and occluded-P fractions. In contrast, the total P had no relationship to the P fractions. In view of these results, the P fractions in the maize rhizosphere and bulky soil were enhanced by phosphorus addition. This suggested that the studied phosphorus forms could influence the uptake of phosphorus by spring maize. On the other hand, the non-rhizosphere soil had higher levels of the different P fractions than the rhizosphere soil (Fig. 4a–e). The amounts of available P were significantly correlated with the concentrations of Ca_2_-P(r = 0.871), Ca_8_-P (r = 0.910), Al-P(r = 0.696), Fe-P(r = 0.759), and occluded-P(r = 0.844) (Table 2). Available phosphorus, soluble phosphorus, DCP-P, occluded-P, Al-P, and Fe-P in non-rhizosphere soil were lower than in rhizosphere soil^27^. For this experiment, a significant difference among the treatments was found only for occluded P. This was in agreement with several studies demonstrating that no-tillage systems were able to maintain higher levels of available P for maize and reduced phosphorus loss^15,51^. Generally, P fractions were dominant in the non-rhizosphere soil under a continuous no-tillage approach.

**Table 2:** Basic physical and chemical properties of the tested soil.

Characteristic	Value
Organic matter	16.97 g kg^−1^
pH	7.10
Available P	34.64 mg kg^−1^
Total P	462.08 mg kg^−1^
Soil bulk density	1.61 g cm^−3^
Soil texture	Black Sandy Loam
Soil type	Black Chernozem

## Materials and Methods

### Experimental design

The experiment was conducted in 2018 in the experimental field of Qingshan Town, Taobei District in Baicheng City, Jilin Province, China (45°41′N latitude, 122°55′E longitude), at an altitude of approximately 200 m. The land use in the plot was a continuous maize monoculture system. This region has a temperate continental monsoon climate with a mean yearly temperature of 5.2 °C and 399.8 mm of average rainfall per annum, and most of the precipitation occurs between April and August. The maize variety used in this region was Xiangyu 998, which is a primary native medium-maize variety. The type of soil was a sandy, loamy chernozem (classified according to the Canadian soil classification system). The chemical properties of the 0–20 cm soil layer are provided in Table 2.

The experiment was conducted over three years (2016–2018) of continuous rotary tillage (CR), continuous no-tillage (CN), plowing-rotary tillage (PR) and plowing-no tillage (PN). There were three replicates per treatment in 12 subplots of 500 m^2^. The crop residues from the previous-year harvest were left in the field as straw return. During soil preparation in the spring, the specifics of the treatments were as follows:(CR) rotary tillage every year, the soil tilling depth was approximately 10–12 cm, no-tillage seeder sowing and fertilizer; (CN)seeder sowing and fertilizer at approximately 20 cm soil depth, direct use of a no-tillage seeder for sowing and fertilization without other treatments;(PR) plowing at a depth of approximately 20 cm in the first year and the same treatment as CR in the last two years; and(PN) plowing and tillage at approximately 20 cm depth in the first year and the same treatment as CN in the second year.

The maize was planted in May at a density of 65000 plants ha^−1^ and was harvested in late October 2018. In each treatment, the combined basal fertilizer applied was (N:P_2_O_5_:K_2_O = 26%:11%:11%) with 800 kg ha^−1^ applied during sowing by a planter machine. Other field management practices were carried out conventionally.

### Soil sampling

Soil sample collection was conducted in May 6, June 24, August 8, October 2, 2018 at four maize growing stages, namely, the seedling stage, elongation stage, tasseling stage, and maturity stage. The samples were divided into rhizosphere soil and non-rhizosphere soil. The soil used for determining the P form was collected at the seedling and maturity stages. Three whole plant roots, including apical and older roots, were dug out from each prominent subplot (at the seedling stage, approximately eight plants were involved). The soil was removed carefully and systematically in a soil area of 28 cm (14 cm on each side of the plant base in the interrows direction) × 35 cm (10 cm in the narrow interrows and 25 cm in the wide interrows) and a depth of 40 cm. Following the careful removal of the unsecured soil from the roots (collected as non-rhizosphere soil), the remaining firmly held earth was shaken gently over a clean paper sheet. After carefully hand-picking out the visible thin roots (except for root hairs), this soil was collected as rhizosphere soil. The collected soil samples were ground down into fine particles and sieved through a 3 mm sieve.

### Soil determination

The soil samples were air-dried, and the P concentration was determined on neutralized extracts using the molybdate-colorimetric method of^52^ at 88 nm. Several consecutive P fractionation procedures have been used to determine the forms of P and distributions of P forms in the soil^53^. Alkaline phosphatase and a series of inorganic forms of soil P (Al-P, O-P, Fe-P,Ca_2_-P, and Ca_8_-P) were determined sequentially according to the method by Jiang and Gu (1989)^54^ as follows:

(i). Soil (1 gram)
  Shake for 1 hour in 50 mL 0.25 mol/L NaHCO_3_ solution (pH = 7.50)
  Centrifuge and remove supernatant for P determination of Ca_2_-P
(ii). Residual from step (i)
  Wash twice with 95% alcohol, shake with 50 mL 0.5 mol/L NH_4_Ac solution (pH = 4.20, let sit for 4 hours, shake again for 1 hour
  Centrifuge and remove supernatant for P determination of Ca_8_-P
(iii). Residual from step (ii)
  Wash twice with saturated NaCl, shake with 50 mL 0.5
  NH_4_F solution for 1 hour
  Centrifuge and remove supernatant for P determination of Al-P
(iv). Residual from step (iii)
  Wash twice with saturated NaCl, shake with 50 mL 0.1 mol/L NaOH-0.1 mol/L Na_2_CO_3_ solution (pH = 8.20) for 2 hours, let sit for 16 hours, shake again for 2 hours
  Centrifuge and remove supernatant for P determination of Fe-P
(v). Wash twice with saturated NaCl, shake with 40 mL 0.3 mol\L sodium citrate solution plus 1gNa_2_S_2_O_6_ heated at 80 °C for 15 min

Centrifuge and remove supernatant for P determination of Occl-P

### Statistical analysis

The data were analyzed, and the differences were compared using SPSS Statistics 23.0 (SPSS, Inc., Chicago, IL, USA). The means were compared by Duncan’s test at the 0.05 significance level. Figures were created in Origin Pro 8.0. The means and standard errors from the statistical analysis were brought into Origin Pro 8.0, and the figures were created using the column tool.

## Conclusion

As the growth period continued, the available P, total P, and P fractions in the soil gradually decreased. The absorption of plant nutrients from rhizosphere soil was high under the continuous no-tillage method. The use of continuous no-tillage methods can effectively increase the content of available nutrients in the soil. The establishment of no-tillage methods can not only increase the amount of available P in the soil but can also effectively maintain a continuous soil P supply throughout the whole maize growth period. The continuous no-tillage treatment promoted the absorption of P nutrients by maize, and the non-rhizosphere soil had sufficient P to exchange with the rhizosphere region for plant uptake.
